# Laparoscopic surgery for colorectal cancers: Current status

**DOI:** 10.4103/0972-9941.28181

**Published:** 2006-12

**Authors:** Parul J Shukla, George Barreto, Piyush Gupta, Shailesh V Shrikhande

**Affiliations:** Department of Gastrointestinal Surgical Oncology, Tata Memorial Hospital, Mumbai, India

**Keywords:** Colorectal cancer, colorectal surgery, laparoscopic surgery

## Abstract

Laparoscopy was introduced more than 15 years ago into clinical practice. However, its role in colorectal surgery was not well established for want of better skills and technology. This coupled with high incidences of port site recurrences, prevented laparoscopic surgery from being incorporated into mainstream colorectal cancer surgery. A recent increase in the number of reports, retrospective analyses, randomized trials and multicentric trials has now provided sufficient data to support the role of laparoscopy in colorectal cancer surgery. We, thus, present a review of the published data on the feasibility, safety, short - and long-term outcomes following laparoscopic surgery for colorectal cancers. While the data available strongly favors the use of laparoscopic surgery in colonic cancer, larger well powered studies are required to prove or disprove its role in rectal cancer.

The morbidity and mortality associated with surgical procedures performed on the colon and rectum have plagued surgeons since time immemorial. As surgeons began to perform open colorectal surgery with increasing confidence these very same problems assumed high significance until the principles of antisepsis and the refinements in surgical technique began to make a considerable dent in these negative outcomes. At this same time, surgeons had already explored the role of minimal invasiveness in treating colorectal problems, viz. the use of the sigmoidoscope to deflate a sigmoid volvulus, perineal procedures for prolapse in elderly persons, etc. The laparoscopic cholecystectomy in 1987 in France, performed by Philipe Mouret for the first time during a laparoscopic gynecologic surgery, rapidly established its role in place of open surgery.[1] However, laparoscopic large bowel surgery did not, for a long time, receive the same degree of acceptance by the surgical community although a few of the initial series had shown promising results. We thus review the problems that were and are still associated with laparoscopic surgery for colorectal cancer while finally attempting to provide an evidence-based review of literature to suggest as to where laparoscopic surgery stands today in the field of colorectal cancers.

## Problems associated with laparoscopic surgery

Laparoscopy for colorectal cancers has not gained universal acceptance for a number of reasons[2,3]

1. Questions on feasibility: Laparoscopic large bowel surgery is much more complicated than laparoscopic cholecystectomy with a steeper learning curve. It requires more advanced laparoscopic technique.
2. Early reports of port-site recurrence / implantation in laparoscopic port sites.[4–8]
3. Presence of an anastomosis or stoma prevents rapid discharge from hospital.
4. The efficacy of large bowel laparoscopic surgery for cancer with respect to factors like tumor clearance and the fear that laparoscopic surgery enhances tumor dissemination.
5. Safety - this implies that laparoscopic surgery carries with it not only some of the general complications encountered with surgery on the bowel, but also a set of unique complications that can occur more or sometimes exclusively only in laparoscopic surgery, e.g., pneumothorax, gas embolism, port site hernias, etc.

## Historical discoveries in the advancement of laparoscopy for colorectal surgery[10]

Advances in techniques of laparoscopy that have paved the way for a shift in the role of laparoscopic surgery in colorectal diseases from the initial intended role of an adjunct to open surgery, to an important operative modality have been, firstly, the realization of the benefit of such a procedure in elderly patients where the morbidity of the large incision of open surgery can be overcome by the reduction in pain and overall bacterial contamination. Videolaparoscopic techniques in colorectal surgery were used for the first time in 1990 by Moises Jacobs in Miami, Florida while performing a right hemicolectomy.[11] The development of a circular stapling device for colostomy closure permitted the first laparoscopic colostomy closure to be performed by in 1990 by Joseph Uddo. The development of a laparoscopic intestinal stapler meant that for the first time, the bowel could be transected intraperitoneally. Dennis Fowler successfully demonstrated this in 1990 when he performed the first laparoscopic sigmoid resection. Subsequent years witnessed more technical innovations that could now make laparoscopic surgeries on the colon and rectum feasible.

## Contraindications to laparoscopic colorectal surgery [Table 1][12]

**Table 1:** Contraindications to laparoscopic colorectal surgery

Cardiovascular or pulmonary instability or failure
Severe or unstable chronic obstructive pulmonary disease or cardiac disease
Coagulopathy not correctable preoperatively
Extreme obesity
Pregnancy
Tumor extensively involving contiguous structures
Diffuse peritoneal contamination with perforated viscus
Acute inflammatory bowel disease (fever, distension, other signs of toxicity) associated with the malignancy
Eneroenteric or enterocutaneous fistula
Multiple previous abdominal surgeries
Obstruction of the intestine with abdominal distension

## RESULTS

### Extent of oncological resection in laparoscopy versus open surgery

The most important aspect if the feasibility of laparoscopy has to be assessed in case of colorectal malignancies is to unconditionally prove that the oncological resection, i.e., the margins of resection - proximal, distal and circumferential and the number of nodes harvested are comparable, if not better than in open surgery.

Numerous studies[2,3,13] in the 1990s had shown that the number of lymph nodes harvested was comparable between open and laparoscopic surgery. Melotti *et al* also concluded that the distance of the tumor from resection margins and the number of lymph nodes harvested with the operative specimen did not vary from those obtained in open surgery.[14] A recent meta-analysis[15] showed that the number of lymph nodes harvested was higher in the laparoscopic group although the differences did not attain statistical significance. Korolija *et al*, however, noted that the difference in distal resection margins (4.6 cm in the lap group versus 5.3 cm in the open group) were statistically significant in favor of open surgery. On the basis of correlation they concluded that laparoscopic surgery was as adequate as the conventional approach. Bretagnol *et al*, have shown that R0 resections could be achieved in 93% of patients undergoing laparoscopic low colorectal and coloanal surgeries.[16]

### Safety and complications

The introduction of laparoscopy into the armamentarium of surgery for colorectal cancer has brought, along with the novel idea of minimal access, a novel set of complications associated with the creation of pneumoperitoneum, port placement and diathermy use that require a considerable amount of skill as well as specialized training if they have to be prevented. Table 2 lists a few of the specific complications associated with laparoscopic colorectal surgery.[17] Six randomized controlled trials[18–23] comparing complication rates did not find any significant difference between laparoscopic and open surgery. In fact, a few did show a lower morbidity in favor of the laparoscopy group. Lacy *et al*[18] concluded that while the operative time was consistently longer in the laparoscopic arm, the peri-operative blood loss as well as morbidity were significantly lower in the laparoscopic arm. Conversion rates vary from 1%[24] to 29%.[25] The conclusion is clear - laparoscopic surgery, if performed by a trained, skillful surgeon, will produce results comparable to open surgery. Poor technique is responsible for the complications encountered and does not reflect an inherent errant potential in laparoscopic surgery. Conversion is not a complication and must be resorted to whenever required.[17]

**Table 2:** Complications of laparoscopic colorectal surgery

Creation of pneumoperitoneum
Gas embolism
Pneumothorax
Cardiac arrhythmia
Impaired venous return
Venous thrombosis
Port placement
Port site recurrence
Hernia
Vessel injury/hemorrhage
Diathermy
Bladder injury
Ureteral injury
Missed lesions
Bowel injury

### Short-term outcomes

Table 3 shows a comparison between various randomized trials comparing short-term outcomes viz, blood loss, analgesic requirement and operative time.

**Table 3:** Comparison of short-term outcomes of laparoscopic and open colorectal surgery

	Operating time (min)		Blood loss		Analgesic requirement	

	Lap	Open	Lap	Open	Lap	Open
Lacy AM[18]	142	118*	105	193*		
Hasegawa H[20]	275	188*	58	137*	Less*	More
Leung KL[21]	189.9	144.2*P<0.001	169	238 *P*=0.06	4.5 (no of inj)	6.9 (no of inj)*P<0.001
Zhou ZG[22]	120	106	20	92*	Less	More
COLOR[23]	145	115*P<0.0001	100	175*P<0.0001	Less*	More
Curet MJ[26]	210	138*	284	407*		
COST Group[27]	150	95*			Less*	More
Sahakitrungruang C[28]	More	Less*				

* Indicates that the difference was statistically significant

Braga *et al*,[29] in their randomized study on short-term outcomes in laparoscopic and open surgery, while analyzing parameters such as wound infection rates, anastomotic leak rates, operative time, blood loss, lymphocyte proliferation in response to Candida albicans and phytohemagglutinin and return to full activity, found that the laparoscopic arm had a significant decrease in the 30-day postoperative morbidity rates compared with the open arm. They also concluded that laparoscopic-assisted colorectal surgeries are associated with better preservation of lymphocyte proliferation indices and gut oxygen tension. Tang *et al*,[30] have shown that there is no difference in the systemic immune response of patients having laparoscopically assisted colectomy and those undergoing conventional open surgery for colorectal cancer. The Cochrane review[31] on the short-term benefits for laparoscopic colorectal resections which analyzed 22 trials and 2965 participants, concluded that whilst the results available favored laparoscopic colorectal resection, only seven of the trials had more than 100 patients. The reviewers believed that the final verdict could only be given after the multicenter trials viz, COLOR, MRC CLASICC and LAPKON II (Germany). The results of the COLOR study[23] have been summarized in Table 3, while the MRC CLASICC[25] has concluded that laparoscopic-assisted surgery for colon cancer is as effective as open surgery and is likely to produce similar long-term outcomes. However, impaired short-term outcomes after laparoscopic-assisted anterior resection (including CRM positivity) for cancer of the rectum do not justify its routine use.

### Long-term outcomes

Laparoscopic colorectal surgeries have constantly been under scrutiny with respect to the long-term outcomes - survival data and recurrence rates. The data available[32–35] has shown long-term survival comparable to conventional open surgery. Local recurrence rates vary from 0-6.6%.[18,36,37] Capusotti *et al*,[33] have even found a better outcome for node - positive patients treated by laparoscopy. Jacob *et al*,[34] have in fact shown better results in the patients undergoing laparoscopic resections. A recent systematic review[38] has shown no difference between laparoscopy and open surgery with regard to the long-term outcome. Other, less powered studies,[39,40] have shown a better outcome for the laparoscopy arm patients. However, multicenter randomized trials are needed to confirm or refute these results.

### Port site recurrence

After the first reported port site metastasis in 1978,[41] numerous studies have been carried out to determine whether laparoscopy is actually associated with an increased incidence of port site recurrences / metastasis. Initial reports showed a high incidence of port site recurrence.[5–9] The possible mechanisms which lead to port site metastasis have been summarized in Table 4.[42] In the review published in 1998, Neuhaus *et al*[42] had strongly suggested an increased incidence of port site metastases due to laparoscopic surgery, warning that in view of the findings, laparcoscopic surgery for colorectal malignancies should occur only within the context of clinical trials. In a prospective randomized controlled trial, Lacy *et al*,[43] found no port site recurrences in the 91 patients studied by them, neither in the laparoscopic nor open surgical arms. Many studies[17,44–46] found a lesser prevalence of port site recurrence than previously shown and realized that the incidence corresponds with wound recurrence seen in open surgery. According to Melotti *et al*,[14] the incidence of port site metastases varies from 0, in the recent studies, to 21.4% in other limited series.[7] Data in support of laparoscopic surgery have steadily increased blaming the initial reports of poor outcome on poor surgical technique. Some policies advised are to avoid contact between laparoscopic instruments and the tumor by bagging and the use of “no - touch” isolation technique suggested by RB Turnbull Jr,[14] meticulous lavage of all wounds with a cytocidal agent,[2] widening the port of extraction of the specimen and use of wound protectors.[17]

**Table 4:** Possible mechanisms which lead to port site metastasis

Mechanical
Direct contamination
Seeding during extraction of tumor through a small wound
Seeding by contact with instruments contaminated with tumor cells
Indirect contamination
Seeding into the wound during episodes of desufflation of the pneumoperitoneum
Cells exist in an aerosol and are transferred to wounds and ports without direct contamination (chimney effect)
Metabolic / immunological
Seeding occurs in both open and laparoscopic wounds, but metastases are more likely after laparoscopy because of locally acting immunological and / or metabolic factors
Hematogenous
Seeding by hematogenous spread during surgery

### Quality of life issues

While the operative time for laparoscopic surgery is obviously more than that for open surgery, there are several beneficial outcomes resulting directly from the use of laparoscopy as compared with open surgery. As there is no large abdominal incision, the corresponding postoperative pain and the ensuing need for analgesia is reduced.[20,23,27,47,48] As the wound is smaller, the likelihood of wound infection is less.[29,49] This attains significance when the patient is a candidate for adjuvant chemotherapy at which time, a wound infection can delay institution of the chemotherapy. The COST study has shown better short-term quality of life. The recurrence and survival rates were equivalent for both groups and for all tumor stages.[27] The median hospital stay and the need for parenteral antibiotics were also shown to be lower in the laparoscopy group. The validity of this shortened hospital stay, though, has been questioned in the light of the stay also being affected by the presence of an anastomosis and the age of the patient.

The incidence of small bowel related problems postoperatively including adhesive obstruction and the incidence of postoperative ventral hernias have also been seen to be on the lower side in the laparoscopically resected group of patients.[50]

The incidence of injuries to the pelvic autonomic nerves during dissection in rectal cancers has been associated with bladder and sexual dysfunction. An increased rate has been noted in some studies.[51–53] This has been attributed possibly to the higher proportion of complete TMEs done by laparoscopy. More trials are required in this aspect.

### Laparoscopy in early lower GI cancers

Laparoscopy has been shown to have an important role in early colonic carcinomas viz, laparoscopic-assisted colonoscopic polypectomy, laparoscopic wedge resection and laparoscopic colostomies with a 67-100% success rate for avoiding a formal bowel resection. This can be achieved by preoperative colonoscopic tattooing for localization.[54]

## CONCLUSION

According to the data available, laparoscopic surgery definitely appears to have a role in colonic malignancies. The short-term and long-term outcomes clearly favor this approach. It should be realized, though, that the benefits of laparoscopic surgery can be obtained only when performed by persons trained in the art of laparoscopy so as to avoid unnecessary morbidity and even the risk of mortality. As for rectal cancer, the present data on the role of laparoscopy is not mature enough, especially for anterior resections. At present, laparoscopic anterior resection should only be considered within the context of clinical trials.

## References

[1] Perrisat J, Vitale GC (1991). Laparoscopic cholecystectomy: Gateway to the future (editorial). Am J Surg.

[2] Psaila J, Bulley SH, Ewings P, Sheffield JP, Kennedy RH (1998). Outcome following laparoscopic resection for colorectal cancer. Br J Surg.

[3] Kwok SP, Lau WY, Carey PD, Kelly SB, Leung KL, Li AK (1996). Prospective evaluation of laparoscopic: Assisted large bowel excision of cancer. Ann Surg.

[4] Cirocco WC, Schartzman A, Golub RW (1994). Abdominal wall recurrence after laparoscopic colectomy for colon cancer. Surgery.

[5] Nduka CC, Monson JR, Menzies-Gow N, Darzi A (1994). Abdominal wall metastases following laparoscopy. Br J Surg.

[6] Prasad A, Avery C, Foly RJ (1994). Abdominal wall metastases following laparoscopy. Br J Surg.

[7] Fusco MA, Paluzzi MW (1993). Abdominal wall recurrence after laparoscopic-assisted colectomy for adenocarcinoma of the colon. Report of a case. Dis Colon Rectum.

[8] Walsh DC, Wattchow DA, Wilson TG (1993). Subcutaneous metastases after laparoscopic resection of malignancy. Aust NZ J Surg.

[9] Ramos JM, Gupta S, Anthone GJ, Ortega AE, Simons AJ, Beart RW (1994). Laparoscopic and colon cancer: Is the port site at risk. A preliminary report?. Arch Surg.

[10] Sgambati SA, Ballantyne GH, Jager RM, Wexner S (1995). Minimally invasive surgery for diseases of the colon and rectum: The legacy of an ancient tradition. Laparoscopic colectomy.

[11] Jacobs M, Verdeja JC, Goldstein HS (1991). Minimally invasive colon resection (laparoscopic colectomy). Surg Laparosc Endosc.

[12] Jenkins NL, Roth JS, Johnson JO, Pofahl WE (2005). Laproscopic colorectal surgery: Indications and techniques. Curr Surg.

[13] Rhodes M, Rudd M, Nathanson L, Fielding G, Siu S, Hewett P (1996). Laparoscopic anterior resection: A consecutive series of 84 patients. Surg Laparosc Endosc.

[14] Melotti G, Tamborrino E, Lazzaretti MG, Bonilauri S, Mecheri F, Piccoli M (1999). Laparoscopic surgery for colorectal cancer. Semin Surg Oncol.

[15] Korolija D, Tadic S, Simic D (2003). Extent of oncological resection in laparoscopic vs. open colorectal surgery: Meta analysis. Langenbecks Arch.

[16] Bretagnol F, Lelong B, Laurent C, Moutardier V, Rullier A, Monges G (2005). The oncological safety of laparoscopic total mesorectal excision with sphincter preservation for rectal carcinoma. Surg Endosc.

[17] Larach SW, Gallagher JT (2000). Complications of laparoscopic surgery for rectal cancer: Avoidance and Management. Semin Surg Oncol.

[18] Lacy AM, Gracia-Valdecasas JC, Delgado S, Castells A, Taura P, Pique JM (2002). Laparoscopic - assisted colectomy versus open coloectomy for treatment of non - metastatic colon cancer: A randomized controlled trial. Lancet.

[19] Winslow ER, Fleshman JW, Birnbaum EH, Brunt LM (2002). Wound complications of laparoscopic versus open colectomy. Surg Endosc.

[20] Hasegawa H, Kabeshima Y, Watanabe M, Yamamoto S, Kitajima M (2003). Randomized controlled trial of laparoscopic versus open coloectomy for advanced colorectal cancer. Surg Endosc.

[21] Leung KL, Kwok SP, Lam SC, Lee JF, Yiu RY, Ng SS (2004). Laparoscopic resection of rectosigmoid carcinoma: A prospective randomized trial. Lancet.

[22] Zhou ZG, Hu M, Li Y, Lei WZ, Yu YY, Cheng Z (2004). Laparoscopic versus open total mesorectal excision with anal sphincter preservation for low rectal cancers. Surg Endosc.

[23] Veldkamp R, Kuhry E, Hop WC, Jeekel J, Kazemier G, Bonjer HJ (2005). Laparoscopic surgery versus open surgery for colon cancer: Short term outcomes of a randomized trial. Lancet Oncol.

[24] Barlehner E, Benhidjeb T, Anders S, Schicke B (2005). Laparoscopic resection for rectal cancer: Outcomes in 194 patients review of literature. Surg Endosc.

[25] Guillou PJ, Quirke P, Thorpe H, Walker J, Jayne DG, Smith AM (2005). Short term end points of conventional versus laparoscopic - assisted surgery in patients assisted colorectal cancer (MRC CLASICC Trial): Multicentre, randomized controlled trial. Lancet.

[26] Curet MJ, Putrakul K, Pitcher DE, Josloff RK, Zucker KA (2000). Laparoscopically assisted and open colectomy for colon carcinoma: Perioperative results and long-term outcome. Surg Endosc.

[27] (2004). Clinical Outcomes of Surgical Therapy Study Group. A comparison of laparoscopically assisted and open colectomy for colon cancer. N Engl J Med.

[28] Sahakitrungruang C, Pattana-Arun J, Tantiphlachiva K, Rojanasakul A (2005). Laparoscopic versus open surgery for rectosigmoid and rectal cancer. J Med Assoc Thai.

[29] Braga M, Vignalli A, Gianotti L, Zuliani W, Radaelli G, Gruarin P (2002). Laparoscopic versus open colorectal surgery: A randomized trial on short - term outcome. Ann Surg.

[30] Tang CL, Eu KW, Tai BC, Soh JG, MacHin D, Seow-Choen F (2001). Randomized clinical trial of the effect of open versus laparoscopically assisted colectomy on systemic immunity in patients with colorectal cancer. Br J Surg.

[31] Schwenk W, Haase O, Neudecker J, Muller JM (2005). Short term benefits for laparoscopic colorectal resection. Cochrane Database Syst Rev.

[32] Lujan HJ, Plasencia G, Jacobs M, Viamonte M, Hartmann RF (2002). Long - term survival after laparoscopiccolon resection for cancer: Complete five - year follow - up. Dis Colon Rectum.

[33] Capusotti L, Massucco P, Muratore A, Amisano M, Bima C, Zorzi D (2004). Laparoscopy as a prognostic factor in curative resection for node positive colorectal cancer: Results for a single - institution nonrandomized prospective trial. Surg Endosc.

[34] Jacob BP, Salky B (2005). Laparoscopic colectomy for colon adenocarcinoma: An 11 - year retrospective review with 5 - year survival rates. Surg Endosc.

[35] Mehta PP, Griffin J, Ganta S, Rangraj M, Steichen F (2005). Laparoscopic - assisted colon resections: Long - term results and survival. JSLS.

[36] Khalili TM, Fleshner PR, Hiatt JR, Sokol TP, Manookian C, Tsushima G (1998). Colorectal cancer: Comparison of laparoscopic with open approaches. Dis Colon Rectum.

[37] Lezoche E, Feliciotti F, Paganini AM, Guerrieri M, De Sanctis A, Minervini S (2002). Laparoscopic vs open hemicolectomy for colon cancer. Surg Endosc.

[38] Reza MM, Blasco JA, Andradas E, Cantero R, Mayol J (2006). systematic review of laparoscopic versus open surgery for colorectal cancer. Br J Surg.

[39] Polliand C, Barrat C, Cahmpault G (2005). Laparoscopic resection of low rectal cancer with a mean follow-up of seven years. Surg Laparosc Endosc Percutan Tech.

[40] Morino M, Parini U, Giraudo G, Salval M, Brachet Contul R, Garrone C (2003). Laparoscopic total mesorectal excision: A consecutive series of 100 patients. Ann Surg.

[41] Dobronte Z, Wittmann T, Karacsony G (1978). Rapid development of malignant metastases in the abdominal after laparoscopy. Endoscopy.

[42] Neuhaus SJ, Texler M, Hewett PJ, Watson DI (1998). Port - site metastases following laparoscopic surgery. Br J Surg.

[43] Lacy AM, Delagado S, Garcia-Valdecasas JC, Castells A, Pique JM, Grande L (1998). Port site metastases and recurrence after laparoscopic colectomy. A randomized trial. Surg Endosc.

[44] Larach SW, Peroza SE, Pantakar SK (1996). Laparoscopic colorectal cancer surgery: Analysis of 5 - years experience. South Med J.

[45] Bouvet M, Mansfield PF, Skibber JM, Curley SA, Ellis LM, Giacco GG (1998). Clinical, pathologic and economic parameters of laparoscopic colon resection for cancer. Am J Surg.

[46] Kim SH, Milsom JW (1998). Is laparoscopic technique oncologically appropriate for colorectal cancer surgery?. J Korean Med Sci.

[47] Milsom JW, Bohn B, Hammerhofer KA, Fazio V, Steiger E, Elson P (1998). A prospective randomized trial comparing laparoscopic versus conventional techniques in colorectal cancer surgery: A preliminary report. J Am Coll Surg.

[48] Stage JG, Schulz S, Moller P, Overgaard H, Andersen M, Rebsdorf-Pedersen VB (1997). Prospective randomized study of laparoscopic versus open colonic resection for adenocarcinoma. Br J Surg.

[49] Davies MM, Larson DW (2004). Laparoscopic surgery for colorectal cancer: The state of the art. Surg Oncol.

[50] Duepree HJ, Senagore AJ, Delaney CP, Fazio VW (2003). Does means of access affect the incidence of small bowel obstruction and ventral hernia after bowel resection? Laparoscopy versus laparotomy. J Am Coll Surg.

[51] Quah HM, Jayne DG, Eu KW, Seow-Choen F (2003). Bladder and sexual dysfunction following laparoscopically - assisted and conventional open mesorectal resection for cancer. Br J Surg.

[52] Rubino F, Leroy J, Marescaux (2003). Bladder and sexual dysfunction following laparoscopically - assisted and conventional open mesorectal resection for cancer. Br J Surg.

[53] Jayne DG, Brown JM, Thorpe H, Walker J, Quirke P, Guillou PJ (2005). Bladder and sexual dysfunction following resection for rectal cancer in a randomized clinical trial of laparoscopic versus open technique. Br J Surg.

[54] Bemelman WA (2005). Minimally invasive surgery for early lower GI cancer. Best Pract Res Clin Gastroenetrol.

